# DYRK1A Protein, A Promising Therapeutic Target to Improve Cognitive Deficits in Down Syndrome

**DOI:** 10.3390/brainsci8100187

**Published:** 2018-10-16

**Authors:** Anis Feki, Youssef Hibaoui

**Affiliations:** 1 Service de gynécologie obstétrique, HFR Fribourg-Hôpital cantonal, Chemin des Pensionnats 2-6, Case Postale, 1708 Fribourg, Switzerland; Anis.Feki@h-fr.ch; 2 Department of Genetic Medicine and Development, University of Geneva Medical School and Geneva University Hospitals, 1 rue Michel-Servet, 1211 Geneva, Switzerland

**Keywords:** down syndrome, trisomy 21, DYRK1A, cognitive impairment, therapy

## Abstract

Down syndrome (DS) caused by a trisomy of chromosome 21 (HSA21), is the most common genetic developmental disorder, with an incidence of 1 in 800 live births. Its phenotypic characteristics include intellectual impairment, early onset of Alzheimer’s disease, congenital heart disease, hypotonia, muscle weakness and several other developmental abnormalities, for the majority of which the pathogenetic mechanisms remain unknown. Among the numerous protein coding genes of HSA21, *dual-specificity tyrosine-(Y)-phosphorylation-regulated kinase 1A* (*DYRK1A*) encodes a proline-directed serine/threonine and tyrosine kinase that plays pleiotropic roles in neurodevelopment in both physiological and pathological conditions. Numerous studies point to a crucial role of DYRK1A protein for brain defects in patients with DS. Thus, DYRK1A inhibition has shown benefits in several mouse models of DS, including improvement of cognitive behaviour. Lastly, a recent clinical trial has shown that epigallocatechine gallate (EGCG), a DYRK1A inhibitor, given to young patients with DS improved visual recognition memory, working memory performance and adaptive behaviour.

## 1. Introduction

Down syndrome (DS), also known as trisomy 21, is caused by an extra copy of chromosome 21 (HSA21). It affects 1 in 800 live births, making DS the most common genetic developmental disorder. Its phenotypic characteristics are complex and variable in penetrance; however intellectual impairment and early onset of Alzheimer’s Disease are common to all individuals with DS [1,2]. Individuals with DS exhibit an impaired development of the nervous system and a delay in the cognitive development leading to mental retardation with an Intelligence Quotient, ranging from 30 to 70 [2,3]. Thus, brains from individuals with DS show a reduced volume and alterations in the number and/or morphology of neurons in several specific regions including the frontal cortex, the hippocampus and the cerebellum [2].

In the past decade, progress has been made in the understanding of how the extra copy of HSA21 contributes to DS phenotype [1]. Among the candidate genes for cognitive impairment in DS, *DYRK1A* which encodes the dual-specificity tyrosine-(Y)-phosphorylation-regulated kinase 1A protein, has received increasing attention, considering its involvement in the neurodevelopment of numerous species [4,5]. In DS individuals, DYRK1A is overexpressed both at the fetal and adult periods, with an approximately 1.5-fold increase in several regions including the frontal, temporal, occipital, and cerebellum [6,7]. *DYRK1A* loss of function is also associated with neurodevelopmental defects (Table 1), as *DYRK1A* haploinsufficiency in human leads to intellectual disability, microcephaly, growth and mental retardation [8,9,10,11,12,13,14,15,16,17,18,19]. Moreover, *Dyrk1a^−/−^* null mutant mice show growth delay and die during midgestation whereas *Dyrk1a*^−/+^ mice display reduced brain size and alterations in the density of neurons in various brain regions (Table 1) [20,21]. In line with this, bacterial artificial chromosome (BAC) transgenic mice carrying an extra copy of *Dyrk1a* show alteration in brain size and neuronal density [22] together with neurodevelopmental delays, motor abnormalities, altered synaptic plasticity, learning and memory deficits (Table 1), thus recapitulating most of the DS phenotype [23,24,25]. Similar phenotypic alterations, albeit with subtle nuances (Table 1), have been also described in studies on different genetically engineered mice including yeast artificial chromosome (YAC) transgenic mice carrying an extra copy of *Dyrk1a* and in mice with partial trisomy (Table 1) [26,27]. 

In order to reduce the activity of DYRK1A, several molecules have been isolated from natural sources and identified as potent in vitro inhibitors such as the plant products harmine and epigallocatechin-3-gallate (EGCG), and the marine sponge product leucettine L41 [4,28,29]. Thereafter, new DYRK1A inhibitors have been developed through drug screening and medicinal chemistry approaches including INDY, FINDY, CX-4945 and ALGERNON [4,28,29,30]. Among those, the green tea polyphenol EGCG is probably the most used in preclinical studies, considering the safety properties of the molecule and the interesting potency to inhibit DYRK1A activity with and IC50 of 330 nM.

Here, we sum up the principal targets of DYRK1A and the possible mechanism of action resulting from DYRK1A inhibition. Thus, the article focuses on the most relevant in vivo preclinical studies evaluating the effect of DYRK1A inhibition in mouse models of DS and more recently in human induced pluripotent stem cells from patients with DS (DS-iPSCs). Lastly, we address the principal outcomes of two clinical trials evaluating the safety and efficacy of EGCG, the only DYRK1A inhibitor tested in patients with DS. 

## 2. DYRK1A Targets and the Possible Mechanisms of Action

**Figure 1:**
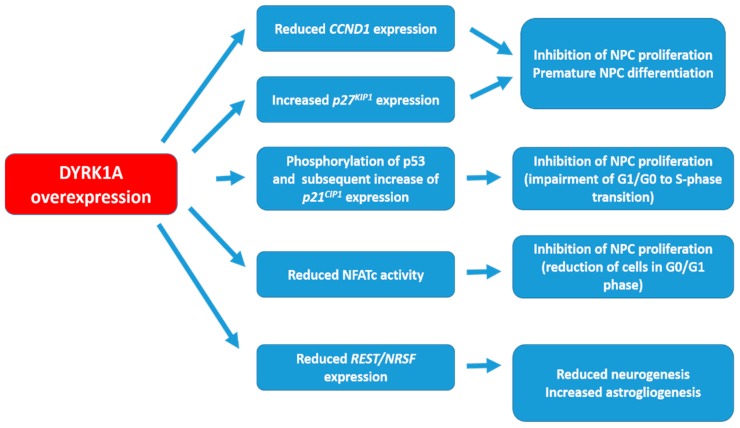
DYRK1A targets and the possible mechanisms underlying neurogenesis impairment in Down syndrome. See text for explanation. CCND1: cyclin D1; NFATc: Nuclear factor of activated T cell cytoplasmic; NPC: neuroprogenitor cell; REST/NRSF: Repressor element-1 binding transcription factor or neuron-restrictive silencer factor.

Several lines of evidence suggest a key role of DYRK1A in the control of cell growth, neurogenesis and neuronal maturation (Figure 1). p53 protein is one of the targets of DYRK1A. In particular, DYRK1A has been shown to phosphorylate p53 leading to the up-regulation of p53 target genes including *p21^CIP1^* (also known as cyclin-dependent kinase inhibitor 1 or CDK-interacting protein 1), a protein involved in cell cycle regulation. The up-regulation of *p21^CIP1^* impairs G1/G0-to-S phase transition, inhibiting neuroprogenitor cell (NPC) proliferation [31,32,33,34]. Consistent with this, increased levels of *p21^CIP1^* have been found in brains from *Dyrk1a* transgenic mice and from fetuses with DS [33]. 

Cyclin D1 (CCND1), a cell cycle protein required for cell proliferation by allowing the entry to the S phase, is also regulated by DYRK1A. In fact, DYRK1A has been shown to phosphorylate cyclin D1 leading to its nuclear export and degradation. There is also evidence that DYRK1A increases G1 duration by reducing cyclin D1 expression [35]. Such mechanisms could explain why *Dyrk1a* overexpression inhibits proliferation and induces premature neuronal differentiation of NPCs [31,32,33,34]. In line with this, overexpression of DYRK1A has been shown to induce the expression of the cyclin-dependent kinase inhibitor *p27^KIP1^* in neural precursors. *p27^KIP1^* further inhibits the cyclin/cyclin-dependent kinase complexes that controls G1/S transition, promoting cell cycle exit and neuronal differentiation [31].

Repressor element-1 binding transcription factor (REST), or neuron-restrictive silencer factor (NRSF), is a transcription factor that plays numerous roles in neurodevelopment including neural lineage specification, synapse formation and function [36,37,38]. Importantly, DYRK1A dosage imbalance can reduce *REST/NRSF* expression by promoting its degradation. Such reduction in DS NPCs has been shown to lead to the subsequent downregulation of important regulators involved in cell adhesion and synapse function [39,40]. Restoring *REST/NRSF* in DS NPCs to near normal levels through DYRK1A inhibition, improves neurogenesis [40]. This improvement likely results from at least in part, an inhibition of the gliogenic shift (i.e., shift from neuronal to glial cells) observed in DS NPCs [40,41].

Moreover, DYRK1A has been shown to phosphorylate the transcription factor NFATc (nuclear factor of activated T cell cytoplasmic), reducing its activity [42]. Therefore, overexpression of DYRK1A in DS leads to a reduction of NFATc transcriptional activity. It has been proposed that another protein resulting from HSA21, RCAN1 (regulator of calcineurin 1 also known as Down syndrome critical region 1, DSCR1) cooperatively interacts with DYRK1A and lead to further dysregulate the NFATc pathway. RCAN1 interacts with and inhibits calcineurin A, a calcium and calmodulin-dependent serine/threonine protein phosphatase that activates NFATc through dephosphorylation. Recent evidence suggests that NFAT regulates the proliferation and differentiation of NPCs [43]. Therefore, the reduced NFATc transcriptional activity triggered by RCAN1 and DYRK1A overexpression might underlie brain-related defects in DS. 

Initially, the overexpression of *APP*, a HSA21 gene encoding the amyloid precursor protein (APP) was thought to confer a higher risk of early onset of Alzheimer’s Disease to patients with DS. Recent results also support a role for DYRK1A in the pathogenesis of Alzheimer’s Disease [44]. DYRK1A has been shown to promote neurofibrillary degeneration directly through hyperphosphorylation of tau and indirectly through phosphorylation of alternative splicing factor. Consistent with this, an increase of the number of DYRK1A-positive and 3R-tau–positive neurofibrillary tangles has been found in brains of patients with DS. Thus, the increased expression of DYRK1A seems to promote brain β-amyloidosis by enhancing the phosphorylation and the amyloidogenic cleavage of APP, increasing the amydogenic levels of Aβ40 and Aβ42 [45]. 

## 3. Preclinical Studies

### 3.1. Results from Mouse Models 

Giving the large body of evidence demonstrating the involvement of DYRK1A in the brain-related defects in patients with DS, many studies have evaluated the effect of DYRK1A inhibition on the learning and memory capacities of mouse models of DS. The Ts65Dn mouse is probably the best characterized and the most used model in preclinical studies for DS. This model carries a segmental trisomy for a distal region of Mmu16 that contains extra copies of several HSA21 genes including *Dyrk1a*. Consequently, Ts65Dn mice exhibited an increase of DYRK1A expression in several brain regions including the cerebellum, the cortex and the hippocampus of mice older than 2.25 months of age; however, this increase was not confirmed in 2.25-month-old Ts65Dn mice (Table 2) [46,47,48,49,50,51,52,53,54]. Alternatively, transgenic mice overexpressing *Dyrk1a* have been used to evaluate the effect of DYRK1A inhibition on brain-related defects. Two different strategies have been essentially used to normalize DYRK1A activity. This was achieved by reducing the expression of DYRK1A through molecular approaches (by viral delivery of short hairpin RNA (shRNA) against *DYRK1A*) or by pharmacological means (with DYRK1A inhibitors) [55,56]. Pharmacological DYRK1A inhibition has been mostly realised through EGCG treatment, with conflicting results having been published regarding the effect of EGCG on cognitive impairment in *Dyrk1a* transgenic mice and in Ts65Dn mice. However, the most studies showed some beneficial effects of EGCG treatment in both transgenic mice overexpressing *Dyrk1a* and in Ts65Dn mice (Table 3).

Treatment of *Dyrk1a* transgenic mic with EGCG (dose of 0.6–1 mg/day and 1.2 mg/day) from gestation to adulthood has been shown to rescue brain morphogenesis alterations (brain weight and volume) and to improve long term memory as revealed by the increase of their performance to near wild-type mice values in the novel object recognition test [57]. Thus, 3-month-old *Dyrk1a* transgenic mice treated with EGCG (dose of 2–3 mg/day) for one month rescued hippocampal neurogenesis by promoting hippocampal cell proliferation [58]. Furthermore, EGCG treatment (120–200 mg/kg/day) of 3-month-old to 4-month-old *Dyrk1a* transgenic mice for 4 weeks, resulted in an improvement of spine density in prefrontal cortex pyramidal neurons together with a normalization of long term potentiation (LTP), a cellular mechanism that underlies learning and memory processes [59]. When Souchet and colleagues tested the effect of one-month treatment of three doses of EGCG (10 mg/kg/day, 60 mg/kg/day and 360 mg/kg/day) in *Dyrk1a* transgenic mice, aged between 3-month-old and 4-month-old, the EGCG dose of 60 mg/kg/day appeared to be the best compromise (i) in enhancing glutaminergic markers without enhancing GABAergic markers expression in the cortex, and (ii) in rescuing behavioural deficits [60]. Moreover, one-month EGCG treatment (9 mg/kg/day) of *Dyrk1a* transgenic mice resulted in an amelioration of hippocampal-dependent spatial and memory performances in the Morris water maze (MWM) test together with a rescue in the novel object recognition (NOR) test [61]. Lastly, normalization of *Dyrk1a* expression in the striatum of *Dyrk1a* transgenic mice through shRNA against *Dyrk1a*, resulted in an attenuation of their hyperactive behavior, a restoration of motor-coordination defects, and an improvement in sensorimotor gating [62].

In 2-month-old Ts65Dn mice, LTP is improved by normalization of *Dyrk1a* expression through shRNA against *Dyrk1a* in the hippocampal region, reaching approximately 50% of the level obtain with wild-type mice. Thus, this normalized initial thigmotaxis but not later thigmotaxis behavior in the MWM test [48]. In line with this, incubation of hippocampal slices from 2-month-old to 6-month-old Ts65Dn mice with 10 µM EGCG corrected LTP to near normal level [65]. In an elegant study, Garcia-Cerro and colleagues crossed Ts65Dn mice with *Dyrk1a* haploinsufficient mice (*Dyrk1a^−/+^*) in order to generate Ts65Dn mice with normalized copy number of *Dyrk1a*. These mice exhibited partial improvements in MWM latency, fear conditioning test and LTP. However, normalizing *Dyrk1a* in these mice did not rescue the density of mature hippocampal granule cells, dentate gyrus volume and subgranular zone area [51]. Moreover, one-month EGCG treatment (9 mg/kg/day) of 2-month-old and 3-month-old Ts65Dn mice improved hippocampal-dependent spatial and memory performances in the MWM test. Thus, this treatment normalized their performance in the NOR test [61]. Despite these promising results, the same group showed that EGCG treatment (30 mg/kg/day) for one month did not improve spatial and memory performances of Ts65Dn mice at the age of the onset of cognitive decline (~6-month-old) [63]. Indeed, only environmental enrichment (EE) alone or EE + EGCG treatment (30 mg/kg/day) improved the hippocampal-dependent learning and memory alterations of 6-month-old Ts65Dn mice. Interestingly, when applying permutation-validated principal component analysis (PCA), the authors found that variables related to the learning improvement explained most of the variance among groups of treatment. In particular, by applying a PCA considering these variables, only “EE + EGCG” treatment significantly ameliorated learning alterations of 6-month-old Ts65Dn mice consistent with the observation that some of the Ts65Dn mice are more prone to treatment efficacy than others. During the same period, Stringer et al. investigated the effect of EGCG treatment (20 mg/kg/day) in Ts65Dn mice from three days after weaning (day 24) to three weeks (day 45, corresponding to adolescence) or seven weeks (day 70, corresponding to adulthood). Importantly, no behavioural improvements were found following EGCG treatment in Ts65Dn mice at both period of age, with respect to the MWM spatial learning tasks, NOR or balance beam tasks [46]. In a more recent study, the same group tested the effect of a higher dose of EGCG (50 mg/kg/day) in 24-day-old Ts65Dn mice for seven weeks and found no behavioural improvements of this dose of EGCG regarding the multivariate concentric square field test, the MWM spatial learning tasks, NOR or balance beam tasks [47]. 

More recently, Nakano-Kobayashi and colleagues have identified and developed new compounds targeting DYRK1A protein. Among those, one called ALGERNON improved brain structure development of Ts1Cje mice, another mouse model of DS. The compound was added to the feed of pregnant DS mice in order to study its impact on brain development of the mouse embryos. Prenatal administration of ALGERNON normalized the thickness of the cortical layer of DS mouse embryos. In addition, this compound promoted neurogenesis of the dentate gyrus of DS mice. Lastly, this treatment improved the cognitive behaviour of DS mice including Y-Maze, Barnes maze spatial memory tasks and fear conditioning tests [30]. 

### 3.2. Results from Human Cells

Considering the difficulties to obtain cells from human brains, only limited studies investigating the role of DYRK1A in DS have been performed in human cell models. In this respect, the discovery that human induced pluripotent stem cells (iPSCs) can be differentiated into neural cells has provided powerful in vitro models for disease modelling and drug screening [66]. Numerous studies have been successful in generating disease-specific iPSCs from patients with DS (reviewed in [67]). Among those, our study was the first pointing to a crucial role of *DYRK1A* in the neurodevelopmental defects of DS [40,68]. Notably, we found that reducing DYRK1A activity to near physiological values through pharmacological approaches (using 10 µM EGCG) or through shRNA against *DYRK1A* improved the number of DS-iPSC-derived NPCs. This improvement was associated with an increase of cell proliferation and a reduction of apoptosis. In the later stages of iPSC differentiation, DYRK1A inhibition by both methods resulted to an increase of the number of neurons derived from DS-iPSCs. This effect was associated with a normalization of important regulators involved in lineage specification, neurogenesis and neuronal maturation including REST/NRSF, NOTCH and WNT signalling [40]. Collectively, not only do these results strongly support the notion that DYRK1A is a key regulator of NPC proliferation and differentiation, but also that it is a rational target for the neurodevelopmental defects of DS. The aforementioned study by Nakano-Kobayashi and colleagues, investigating the effect of the DYRK1A inhibitor ALGERNON in NPCs derived from DS-iPSCs, is an excellent illustration of this [30]. 

## 4. Clinical Studies

Up to now, most of the therapies used to improve cognition in DS are of two types. The first type is using neurotransmitter-based strategies that were initially implemented for Alzheimer’s Disease such as acetylcholine esterase inhibitors (donezepil), GABAergic antagonists (pentetrazol) and N-methyl-D-aspartate receptor antagonists (memantine). The second are vitamin-based or mineral supplement-based therapies (Reviewed in [55,56]).

In light of the encouraging preclinical data reported by our group and others, which provide compelling evidence for the role of DYRK1A signalling in the cognitive impairment in DS, it has indeed become a viable option to initiate testing of DYRK1A inhibitors in the clinical setting. To our best knowledge, only two clinical trials targeting DYRK1A in patients with DS have been published. In a pilot study, 31 young adults with DS (aged 14 to 29 years) were enrolled in a randomized, double-blind study to test EGCG (oral dose of 9 mg/kg/day) or placebo treatments over a period of 3 months (study participants were also followed up for 3 months after treatment discontinuation). EGCG treatment was shown to improve visual recognition memory, working memory performance, psychomotor speed and social functioning. Notably, these improvements were moderate and disappeared within the 3-month post-treatment period. Most importantly though, the study established favourable safety profile of EGCG treatment in young individuals with DS. In this regard, EGCG showed no alteration of the hepatic function (as measured by the activity of aspartate transaminase and alanine transaminase) together with an improvement of lipid profile (including total and LDL cholesterol) [61].

In a second clinical study, the same group evaluated the safety and efficacy of cognitive training with EGCG supplementation versus cognitive training alone in a double-blind, randomised, placebo-controlled, phase 2 trial (TESDAD study) for 12 months. 84 young adults with DS aged from 16 to 34 years old were enrolled and assigned to cognitive training alone or cognitive training with EGCG supplementation (oral dose of 600 mg/day for participants weighting 50–75 kg or EGCG 800 mg/day for participants weighting 75–100 kg). Regarding the first outcome measures of this clinical trial, 13 of 15 tests of the TESDAD battery and 8 of 9 adaptive skills in the Adaptive Behaviour Assessment System II (ABAS—II) were not significantly different between the two groups. For the combined EGCG treatment with cognitive training, improvements in visual recognition memory, inhibitory control and adaptive behaviour were demonstrated. Lastly, for the secondary outcome measures of this clinical study, neuroimaging analysis revealed improvements in functional connectivity and normalisation of cortical excitability when combined EGCG treatment and cognitive training [69]. 

## 5. Conclusions and Challenges

While the vast majority of the preclinical studies performed in mouse models of DS have reported improved behavioural outcomes with EGCG supplements, two studies from the same group have shown that EGCG, even at high doses, does not improve behavioural outcome of Ts65Dn mice (Table 3). Several factors can account for these discrepancies: (i) the composition of the treatment (pure EGCG vs. EGCG in combination with other green tea extracts); (ii) EGCG dosage; (iii) route of administration; (iv) duration of the treatment; (v) age of the mice which reflect directly the extent of cognitive impairment (i.e., the onset of cognitive decline in Ts65Dn mice is at 6-months); (vi) species and strain; (vii) methods used to evaluate cognitive endpoints. All these conditions will inexorably impact the outcomes and comparability of the studies. 

Even if most of the pharmacotherapies have been performed in adult mice, more recent ones conducted at the neonatal and prenatal stages suggest that early interventions are more efficacious in rescuing brain-related defects of DS [30,56,64]. In this respect, it remains crucial to understand with precision the spatial and temporal expression of DYRK1A during neurodevelopment (see Table 2), in order to adapt the treatment with DYRK1A inhibitors (dosage, beginning and duration of the treatment). Considering that *DYRK1A* haploinsufficiency is associated with reduced brain size and neurodevelopmental delays in human [8,9] and in mice [20,21] (Table 1), reducing DYRK1A activity below the physiological levels would suggest deleterious effects for neurodevelopment.

Also, there is a real need to develop novel biomarkers that are minimally invasive and that can be used to evaluate DYRK1A expression/activity in the brain with a high degree of sensitivity and specificity. Such biomarkers should also serve to predict and assess the efficacy of treatments. Previous studies have shown that plasma homocysteine level correlates with Dyrk1a expression [6,70]; it has been shown that Dyrk1a overexpression increased the hepatic NAD(P)H:quinone oxidoreductase and S-adenosylhomocysteine hydrolase activities, decreasing plasma homocysteine level [70]. Therefore, De la Torre and colleagues measured plasma homocysteine levels as an efficacy biomarker for DYRK1A activity in human. They showed that plasma homocysteine level changes were correlated with Dyrk1a expression/activity of mouse hippocampus. One-month EGCG treatment (9 mg/kg/day) normalized DYRK1A activity in the hippocampus and plasma homocysteine levels of *Dyrk1a* transgenic mice. Similarly, it normalized plasma homocysteine levels in Ts65Dn mice [61]. However, these findings necessitate further studies to confirm the correlation between Dyrk1a expression/activity in the brain and plasma homocysteine levels. It will be of interest to confirm this correlation using for instance other DYRK1A inhibitors. 

To date, most of the insights regarding brain-related defects of DS have been gained through transgenic mouse models. Despite similarities with human phenotype, they have several drawbacks and cannot integrate the specificities of human diseases. The discovery that human iPSCs can be differentiated into neural cells has allowed new opportunities for DS modelling, drug screening and perhaps for regenerative medicine purposes [66]. Over the past decade, several laboratories have succeeded in generating DS-iPSCs from patients with DS [67]. Brain-related defects are probably the most studied phenotype in iPSC-based models. Regarding the DYRK1A protein and its potential for use as a drug target, only two studies have investigated the effect of its inhibition on neuronal development of DS-iPSCs [30,40]. Reducing DYRK1A activity to near normal values enhanced neurogenesis of NPCs derived from DS-iPSCs and the density of neurons [30,40]. Such iPSC-based models are likely to enable important advances both in fundamental biology and in drug discovery [66]. Over the last eight years, numerous studies have demonstrated that iPSCs generated for disease modelling purposes have resulted in the identification of novel therapeutic targets in a relevant cellular model (human cells and tissue of interest for the disease) and at the same time, enable testing for drug efficacy and safety at preclinical stages [71]. When one considers the alarming rate of attrition of drug candidates in clinical development programmes for diseases affecting the central nervous system [72,73], it is hoped that results obtained with DS-iPSC-derived cells will provide a welcome relief of this area of research. 

In conclusion, the promising results obtained with mouse models of DS and more recently in human DS-iPSCs, provides good reason for optimism that DYRK1A inhibition may well be a relevant target to improve cognition of patients with DS. 

## Figures and Tables

**Table 1:** *DYRK1A* aneuploidies or mutations in human and mice.

Model	Species	*Dyrk1a* Aneuploidies or Mutations	Phenotypes	References
mBACtgDyrk1a	Mouse	Triplication of the mouse *Dyrk1a* gene	Alterations in brain size and neuronal density. Neurodevelopmental delays, motor abnormalities, altered synaptic plasticity, learning and memory deficits.	[22,23,24]
YACtg152F7 (versus YACtg141G6)	Mouse	Yeast artificial chromosome containing *PIGP*, *TTC3*, *DSCR9*, *DSCR3* and *DYRK1A* (for YACtg152F7) versus YAC containing *PIGP*, *TTC3*, *DSCR9*, *DSCR3* but not *DYRK1A* (for YACtg141G6)	Reduced performance in Morris water-maze and fear-conditioning tests consistent with learning and memory defects. Normal hippocampal long term potentiation.	[26]
Dyrk1a^−/−^	Mouse	Loss of function of *Dyrk1a*	Mid-gestational death (between E10.5 and E13.5 periods). Before death, embryos showed reduction of brain size (30%), growth retardation, morphological developmental delay in the primitive organs.	[21]
Dyrk1a^−/+^	Mouse	*Dyrk1a* haploinsufficiency	Reduced brain size and alterations in the density of neurons in various brain regions. The pyramidal cells from the cortex are smaller, with less branching and dentritic spines. Decreased viability, pre- and post-natal growth retardation, developmental delays, motor and learning difficulties. Atypical behaviors including increased anxiety, impaired reactivity to stress.	[20,21]
152F7, 230E8, 141G6, 285E6 and Ts65Dn	Mouse	Segmental trisomies 21 produced by inserting human contiguous fragments in the D21S17-ETS2 region of HSA21	Only the 152F7 mouse strain which contains a triplication of *Dyrk1a*, is closer to Ts65Dn mice for reference memory: learning slope and probe test in the Morris water maze. The other cognitive processes such as working, discriminating and episodic memory are not affected in the 152F7 mice.	[27]
Individuals with *DYRK1A* haploinsufficiency	Human	*DYRK1A* haploinsufficiency resulting from deletions, translocations, frameshift, splice site, nonsense, misense in *DYRK1A* gene	Intellectual disability, microcephaly, autism spectrum disorder, speech and motor delays, gait disturbances, facial dysmorphology and short stature is common to all individuals. Seizures, feeding difficulties, vision abnormalities and intrauterine growth restriction are present in ~2/3 of all individuals.	[8,9,10,11,12,13,14,15,16,17,18,19]

**Table 2:** Dyrk1A expression (or activity) in brain regions at different ages of Ts65Dn mice.

Age of Ts65Dn Mice	Brain Regions	DYRK1A Expression or Activity	References
2.25 month	Cerebellum, Hippocampus	1.2-fold increase of Dyrk1a activity but not significant 1.4-fold increase of Dyrk1a activity but not significant	[46]
2.25 month	Cerebellum, Cortex, Hippocampus	Decreased Dyrk1a protein expression (−60%) No difference in Dyrk1a expression No difference in Dyrk1a expression	[47]
3.5 month	Hippocampus	Increased Dyrk1a protein expression (+26%)	[48]
4.4–7.8 month	Cerebellum, Cortex, Hippocampus	Increased Dyrk1a protein expression (+24%) Increased Dyrk1a protein expression (+58%) Increased Dyrk1a protein expression (+31%)	[50]
5–6 month	Cerebellum, Hippocampus	Increased Dyrk1a protein expression (+60%) Increased Dyrk1a protein expression (+58%)	[51,52]
5–6 month	Cerebellum, Cortex, Hippocampus	Increased Dyrk1a protein expression (+60.3%) Increased Dyrk1a protein expression (+64.3%) Increased Dyrk1a protein expression (+68%)	[53]
~6 month	Cerebellum, Cortex, Hippocampus	Increased Dyrk1a protein expression (+22%) Increased Dyrk1a protein expression (+58%) Increased Dyrk1a protein expression (+30%)	[49]
7–8 month	Cortex, Hippocampus	Increased Dyrk1a protein expression (+32%) Increased Dyrk1a protein expression (+41%)	[54]
~12 month	Cerebellum, Cortex, Hippocampus	Increased Dyrk1a protein expression (+98%) Increased Dyrk1a protein expression (+98%) Increased Dyrk1a protein expression (+100%)	[49]

**Table 3:** Preclinical studies showing the effects of normalizing DYRK1A expression (through genetic approaches) or DYRK1A activity (through EGCG treatment) in mouse and human models of DS.

Model	Species	Treatment, Intervention	Effect on Brain Structures and Behavior	References
TgDyrk1a	Mouse	Normalisation of Dyrk1a through *Dyrk1a* shRNA in the striatum of 2–3 month-old mice.	Attenuation of the hyperactive behavior, improvement of motor coordination (treadmill test) and PPI (prepulse inhibition) of startle reflex.	[62]
TgDyrk1a	Mouse	Decaffeinated MGTE in drinking water (EGCG concentration of 90 mg/mL for a dose of 2–3 mg/day) for 1 month in 3 week-old mice.	Improvement of hippocampal cell proliferation.	[58]
TgDyrk1a	Mouse	MGTE lightly caffeinated (45% EGCG) in drinking water (EGCG concentration of 90 mg/mL for a dose of 2–3 mg/day) for 1 month in 3 month-old mice.	Improvement of the MWM spatial learning tasks and NOR test.	[61]
mBACtgDyrk1a	Mouse	Green tea extract (45% EGCG) in drinking water with an equivalent dose of 120–200 mg/kg/day EGCG, for 4–6 weeks in 3–4 month-old mice.	Improvement of spine density in prefrontal cortex pyramidal neurons and normalization of LTP.	[59]
mBACtgDyrk1a	Mouse	MGTE lightly caffeinated (45% EGCG) in food supplementation (EGCG dose of 10 mg/kg/day or 60 mg/kg/day or 360 mg/kg/day) for 4 weeks in 3–4 month-old mice.	60 mg/kg/day appeared to be the best compromise in enhancing glutaminergic markers without enhancing GABAergic markers expression in cortex. Rescue of glutaminergic markers expression (but not of GABAergic markers) with all doses in hippocampus. Improvement of the rate of spontaneous alternation.	[60]
YACtg152F7	Mouse	Green tea in drinking water with an equivalent dose of 0.6–1 mg/day EGCG or polyphenon 60 with an equivalent dose of 1.2 mg/day from gestation to adulthood.	Rescue of brain weight and volume (and volume of hypothalamus/thalamus). Improvement of NOR test.	[57]
Ts65Dn	Mouse	Normalisation of *Dyrk1a* through *Dyrk1a* shRNA in the hippocampus.	Improvement of LTP and initial thigmotaxis but not later thigmotaxic behavior. No improvement of MWM Latency.	[48]
Ts65Dn	Mouse	Ts65Dn crossed with Dyrk1a^+/−^ mice.	Improvement of the MWM, fear conditioning test and LTP. Do not improve the density of mature hippocampal granule cells, dentate gyrus volume and subgranular zone area. Do not rescue behavioral alterations (hyperactivity/attention).	[51]
Ts65Dn	Mouse	MGTE lightly caffeinated (45% EGCG) in drinking water (EGCG concentration of 90 mg/mL for a dose of 2–3 mg/day) for 1 month in 3 month-old mice.	Improvement of the MWM spatial learning tasks and NOR test.	[61]
Ts65Dn	Mouse	Decaffeinated MGTE in drinking water (EGCG dose of 30 mg/kg/day) for 1 month in 5–6 month-old mice.	No improvement in spatial and memory performance. Improvement of the Gallagher index and the thigmotaxis along learning sessions (but no improvement in the latency to reach the escape platform).	[63]
Ts65Dn	Mouse	Pure EGCG in drinking water at ~20 mg/kg/day starting from 24 days of age for 3 or 7 weeks.	No improvement in the MCSF, the MWM spatial learning tasks, NOR or balance beam tasks.	[46]
Ts65Dn	Mouse	Pure EGCG in drinking water at ~50 mg/kg/day starting from 24 days of age for 7 weeks.	No improvement in the MCSF, the MWM spatial learning tasks, NOR or balance beam tasks.	[47]
Ts65Dn	Mouse	Polyphenon 60 * in drinking water at 225 mg/kg/day, containing 27% EGCG (~60mg/kg/day) for 6 weeks in 3–4 month-old mice.	Rescue of GABAergic and glutaminergic markers expression in the cortex and the hippocampus (but not in the cerebellum). Improvement in the Y-maze test.	[60]
Ts65Dn	Mouse	Pure EGCG in drinking water at ~25 mg/kg/day starting from postnatal day 3 to postnatal day 15.	Improvement of the proliferation and connectivity in neocortex and hippocampus at P15. However, these improvements measured at P15 disappeared at P45. No improvements in Y-maze and MWM at P45.	[64]
NPCs and neurons derived from DS-iPSCs	Human	Normalisation of DYRK1A through *DYRK1A* shRNA or treatment with 10 µM EGCG of NPCs and neurons derived from DS-iPSCs.	Improvement of proliferation and decrease of apoptosis of NPCs derived from DS-iPSCs. Rescue of neurogenesis impairment of NPCs and neurons derived from DS-iPSCs. Improvement of *REST/NRSF*, *NOTCH* and *WNT* signaling in NPCs derived from DS-iPSCs.	[40]
NPCs derived from DS-iPSCs	Human	Treatment of NPCs derived from DS-iPSCs with 5µM ALGERNON (#688).	Increased of proliferation of NPCs derived from DS-iPSCs and increased proportion of these NPCs in G1-phase.	[30]

ALGERNON #688: « altered generation of neurons » compound with a potency to inhibit DYRK1A activity with an IC50 of 76.9 nM; DS-iPSCs: induced pluripotent stem cells derived from patients with DS; EGCG: epigallocatechine gallate; LTP: long-term potentiation; NOR: novel object recognition; NPCs: neuroprogenitor cells; MCSF: multivariate concentric square field; MGTE: Mega Green Tea Extract; MWM: Morris water maze; TgDyrk1A: transgenic *Dyrk1a* mice. * POLYPHENON 60 contains in addition to 27% EGCG, 42% of other catechins including epicatechine, epicatechine gallate, epigallocatechine and gallocatechine (with no effect on DYRK1A). For clarity, only studies using Ts65Dn model are presented in this table.

## Abbreviations

DS: Down syndrome; DS-iPSCs: iPSCs carrying trisomy 21 anomaly; DYRK1A/Dyrk1a: dual-specificity tyrosine-(Y)-phosphorylation-regulated kinase 1A; EGCG: epigallocatechin-3-gallate; HSA21: human chromosome 21; LTP: long-term potentiation; MCSF: multivariate concentric square field; MWM: Morris water maze; NOR: novel object recognition; NPCs: neuroprogenitor cells; TgDyrk1a: transgenic *Dyrk1a* mice.
